# Association between triglyceride-glucose index and in-hospital mortality in critically ill patients with sepsis: analysis of the MIMIC-IV database

**DOI:** 10.1186/s12933-023-02041-w

**Published:** 2023-11-08

**Authors:** Rui Zheng, Songzan Qian, Yiyi Shi, Chen Lou, Honglei Xu, Jingye Pan

**Affiliations:** 1 Department of Critical Care Medicine, Sir Run Run Shaw Hospital, School of Medicine, Zhejiang University, Hangzhou, Zhejiang China; 2 Department of Intensive Care Unit, The First Affiliated Hospital of Wenzhou Medical University, Wenzhou, 325000 China; 3 Department of Anesthesiology, The First Affiliated Hospital of Wenzhou Medical University, Wenzhou, 325000 China; 4 School of the First Clinical Medical Sciences, Wenzhou Medical University, Wenzhou, 325000 China; 5 Key Laboratory of Intelligent Treatment and Life Support for Critical Diseases of Zhejiang Provincial, Wenzhou, 325000 Zhejiang People’s Republic of China; 6 Zhejiang Engineering Research Center for Hospital Emergency and Process Digitization, Wenzhou, 325000 Zhejiang China

**Keywords:** Triglyceride-glucose index, In-hospital mortality, Sepsis, MIMIC-IV database

## Abstract

**Background:**

This study aimed to explore the association between the triglyceride-glucose (TyG) index and the risk of in-hospital mortality in critically ill patients with sepsis.

**Methods:**

This was a retrospective observational cohort study and data were obtained from the Medical Information Mart for Intensive Care-IV (MIMIC IV2.2) database. The participants were grouped into three groups according to the TyG index tertiles. The primary outcome was in-hospital all-cause mortality. Multivariable logistics proportional regression analysis and restricted cubic spline regression was used to evaluate the association between the TyG index and in-hospital mortality in patients with sepsis. In sensitivity analysis, the feature importance of the TyG index was initially determined using machine learning algorithms and subgroup analysis based on different subgroups was also performed.

**Results:**

1,257 patients (56.88% men) were included in the study. The in-hospital, 28-day and intensive care unit (ICU) mortality were 21.40%, 26.17%, and 15.43% respectively. Multivariate logistics regression analysis showed that the TyG index was independently associated with an elevated risk of in-hospital mortality (OR 1.440 [95% CI 1.106–1.875]; P = 0.00673), 28-day mortality (OR 1.391; [95% CI 1.52–1.678]; P = 0.01414) and ICU mortality (OR 1.597; [95% CI 1.188–2.147]; P = 0.00266). The restricted cubic spline regression model revealed that the risks of in-hospital, 28-day, and ICU mortality increased linearly with increasing TyG index. Sensitivity analysis indicate that the effect size and direction in different subgroups are consistent, the results is stability. Additionally, the machine learning results suggest that TyG index is an important feature for the outcomes of sepsis.

**Conclusion:**

Our study indicates that a high TyG index is associated with an increased in-hospital mortality in critically ill sepsis patients. Larger prospective studies are required to confirm these findings.

**Supplementary Information:**

The online version contains supplementary material available at 10.1186/s12933-023-02041-w.

## Introduction

Sepsis, a condition characterized by a dysregulated immune response to infection, is a prominent contributor to global mortality [1]. Despite ongoing efforts, both the incidence and mortality of sepsis have demonstrated limited reductions over the past decades [2, 3]. This further emphasize the importance of identifying risk factors associated with sepsis outcomes, enabling early prevention strategies.

Insulin resistance (IR) is a pathological physiological state characterized by diminished sensitivity in the peripheral tissues to insulin, which is often prevalent in patients with sepsis and manifests as elevated insulin levels and reduced sensitivity. Acute glucose fluctuations may increase mortality risk in patients with sepsis [4]. The triglyceride–glucose (TyG) index has emerged as a surrogate marker of insulin resistance, some studies indicated that TyG index is associated with the progression of metabolic disorders [5, 6]. The extensive release of inflammatory factors and imbalanced oxidative stress are likely culprits for inducing insulin resistance in the context of sepsis. As such, it is imperative to further explore the association between the degree of insulin resistance and the prognosis of septic patients.

A multitude of cross-sectional and retrospective studies have revealed noteworthy links between the TyG index and all-cause mortality among critically ill patients afflicted with conditions such as ischemic stroke, chronic kidney disease, and cardiac arrest [7–9]. Currently, research investigating the connection between TyG index and sepsis outcomes in the literature is lacking. This study aimed to investigate the association between the TyG index and the clinical outcome of patients with sepsis, ultimately revealing the profound implications of insulin resistance on sepsis.

## Methods

### Study population

This was a retrospective observational cohort study with longitudinal follow-up of patients. Medical Information Mart for Intensive Care-IV (MIMIC-IV-2.2) is a freely accessible database that encompasses over 50,000 ICU admissions at the Beth Israel Deaconess Medical Center in Boston, Massachusetts, from 2008 to 2019 [10]. The MIMIC-IV database contains a wealth of information, including demographics, vital signs, test results, and diagnoses categorized using codes from both the International Classification of Diseases, Ninth Revision (ICD-9) and Tenth Revision (ICD-10). To access this database, one of the authors (Rui Zheng) obtained the necessary certification and subsequently extracted the relevant variables for our study (certification number: 1797305). Individual patient consent was not needed due to the anonymized nature of the patient health information within this database.

**Fig. 1:**
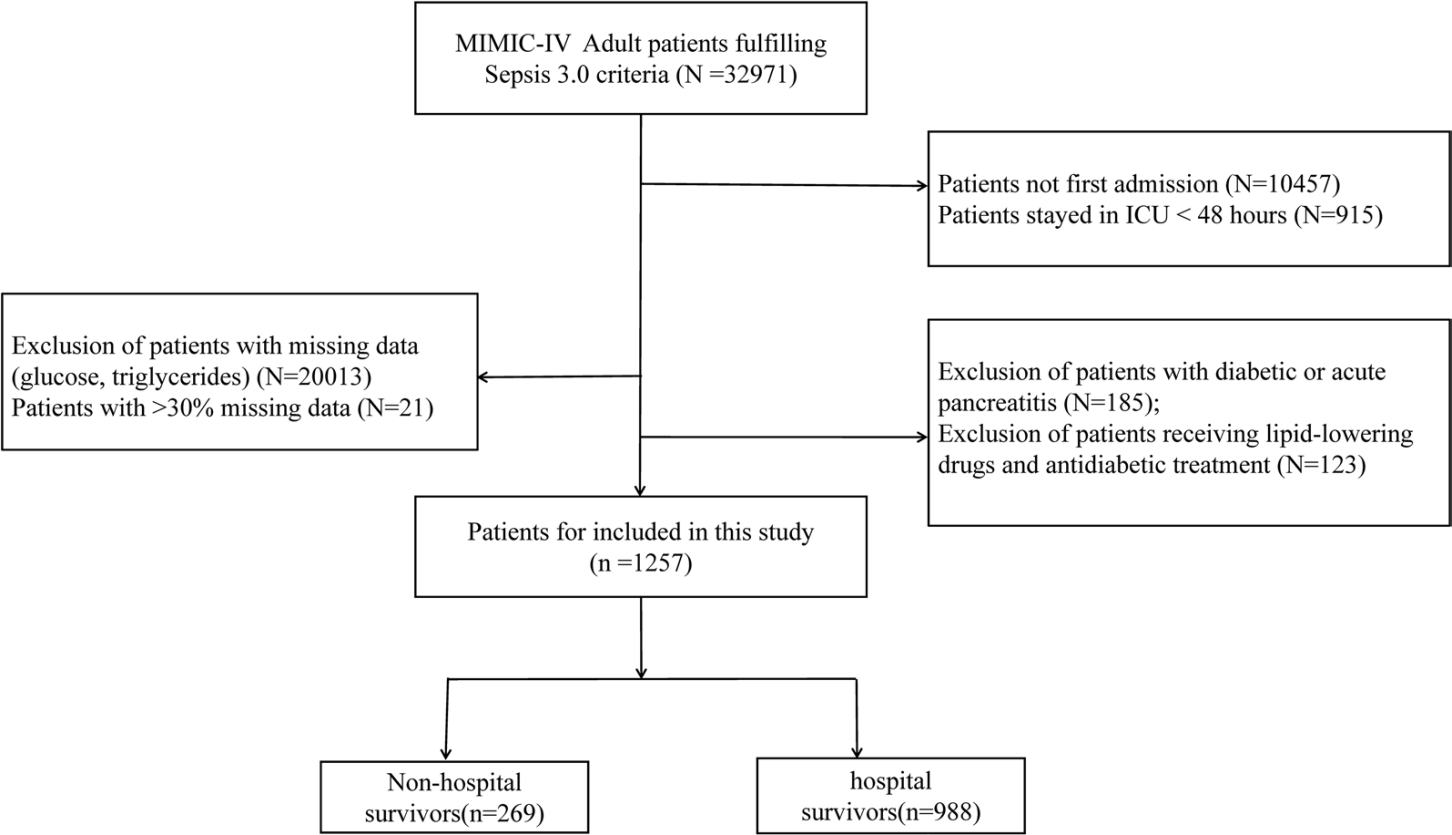
Inclusion/exclusion criteria. MIMIC: Medical Information Mart for Intensive Care

Patients diagnosed with sepsis according to the sepsis 3.0 diagnostic criteria were included in this study, which with infection and Sequential Organ Failure Assessment (SOFA) score ≥ 2 [11]. The method for obtaining patients with sepsis who meet the Sepsis 3.0 diagnostic criteria from the MIMIC database is consistent with previously published studies[12], details in Additional file 1-A. The exclusion criteria were as follows: (1) patients stayed in ICU < 48 h; (2) multiple admissions to the ICU for sepsis, for whom only data from the first admission were extracted; (3) insufficient data (such as triglycerides and fasting blood glucose); (4) patients with diabetic or acute pancreatitis; (5) patients receiving lipid-lowering drugs and antidiabetic treatment (Fig. 1).

### Variable extraction

In this study, the information was extracted using PostgresSQL software (version 13.7.2) and Navicat Premium software (version 16) through the execution of a Structured Query Language (SQL). Data extracted from the MIMIC-IV database on the first 24 h of ICU admission included age, Gender, Body mass index (BMI), and SOFA score and each component of the SOFA score. Other relevant data, including laboratory test results, clinical outcomes, and comorbidities were obtained. All laboratory parameters extracted from the MIMIC-IV (2.2) database were measured on the first time after ICU admission. Follow-up started on the ICU admission date and ended on the date of death. The TyG index was calculated using the formula ln [fasting triglycerides (mg/dl) × fasting blood glucose (mg/dl)/2]. Low HDL (high-density lipoprotein) was defined as HDL-C < 40 mg/dL for males or < 50 mg/dL for females. AKI (acute kidney injury) were defined by both serum creatinine (Scr) and the volume of urine during the first 48 h after ICU admission. NLR (neutrophil to lymphocyte ratio) was defined as neutrophil to lymphocyte ratio.

There is no consensus regarding the standard percentage of missing values for excluding variables from the analysis. For our study, we set our threshold at 60%, considering that Zhang et al. [13] omitted variables with over 70% missing values in their analysis. Before each model fitting process, we assumed missing data were “missing at random” (MAR) [14, 15]. The “missForest” package in R studio was employed to impute the data [16, 17].

### Primary outcome and secondary outcomes

The primary outcome of the present study was in-hospital all-cause mortality, and the secondary endpoints was ICU mortality and mortality within 28 days after admission to the ICU. Patient mortality information for discharged patients was accessed from the US Social Security Death Index.

### Feature selection

Before investigating the association between the TyG index and in-hospital mortality in sepsis patients, we first employed machine learning algorithms for feature selection to determine their importance in the prognostic model. In this regard, a key approach is the Boruta algorithm, which is a widely utilized feature-selection method. The essence of this algorithm is based on two concepts: "shadow features" and "binomial distribution.". Boruta generated a set of feature replicas, referred to as shadow features, from the original dataset. If a feature's Z-score surpasses the maximum possible Z-score for shadow features, it is considered significant and retained; otherwise, it is excluded [18]. Additionally, we employed a random forest model for variable feature selection and employed the SHapley Additive extension (SHAP) package to visualize variable importance [19]. The SHAP package, implemented via the SHAP Python package (version 0.39.0), facilitates model interpretation to mitigate the inherent black-box nature of machine learning, thereby assisting clinicians in comprehending the outcomes provided by the models [20].

### Statistical analysis

**Fig. 2:**
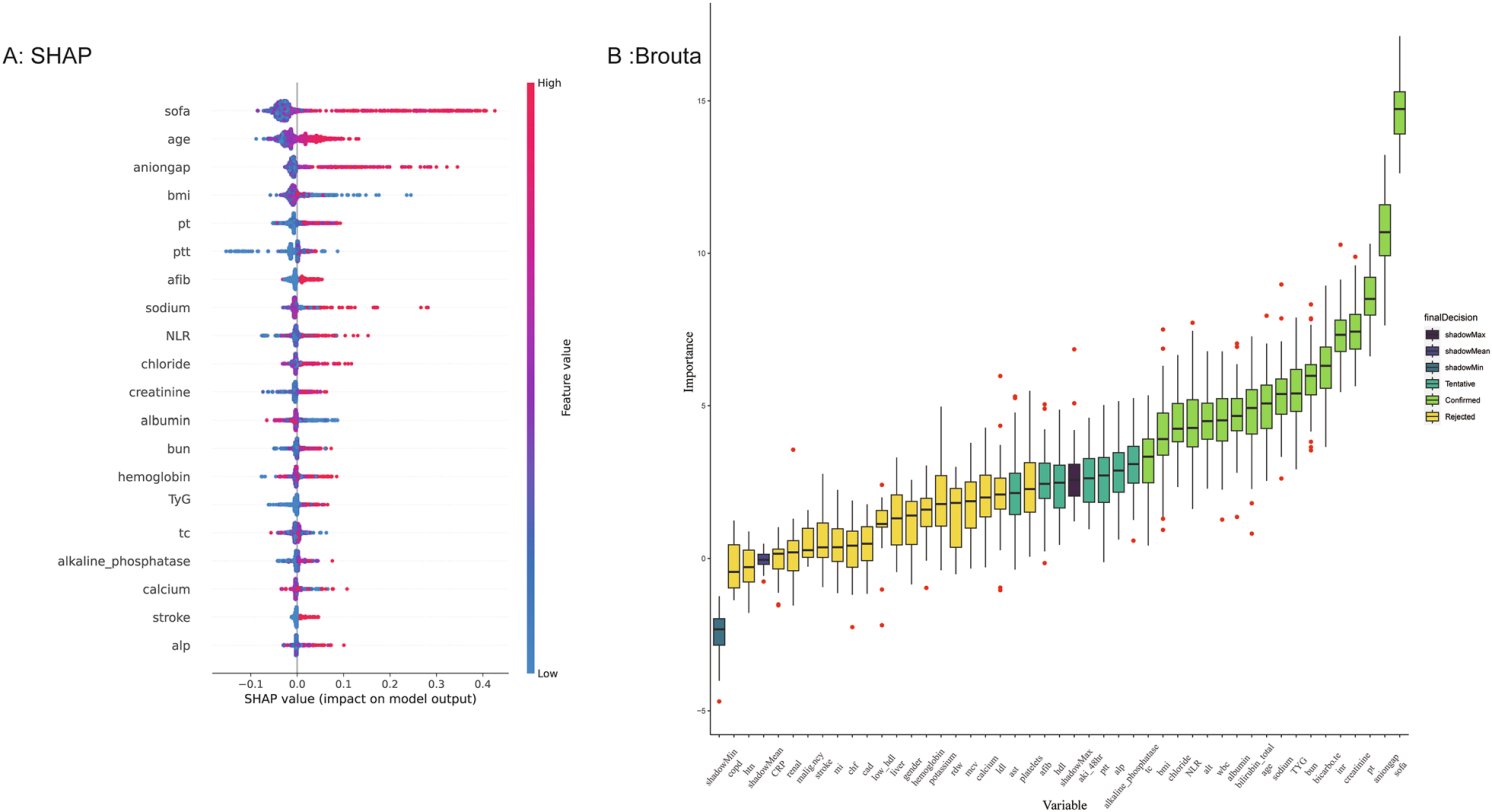
Application of Machine Learning in Feature Selection. **A** Shapley Additive Explanations (SHAP) for the random forest model. **A** Distribution of the impact of each feature on the model output. Each dot represents a patient in a row. The colors of the dots represent the feature values: red represents larger values and blue represents lower values. **B** Feature selection for the relationship between various TyG indices and in-hospital mortality was analyzed using the Boruta algorithm. **B** The horizontal axis shows the name of each variable, whereas the vertical axis represents the Z-value of each variable. The box plot depicts the Z-value of each variable in the model calculation, with green boxes representing important variables, blue boxes representing tentative attributes, and yellow boxes representing unimportant variables. SOFA: Sequential Organ Failure Assessment; Activated Partial Thromboplastin Time WBC: White Blood Cell Count BMI: Body Mass Index LDL: Low-Density Lipoprotein PT: Prothrombin Time ALP: Alkaline Phosphatase RDW: Red Cell Distribution Width HDL: High-Density Lipoprotein. TyG: triglyceride glucose index ALT: Alanine Aminotransferase; COPD: Chronic Obstructive Pulmonary Disease CAD: Coronary Artery Disease; CHF: Congestive Heart Failure; HTN: hypertension

Categorical variables were evaluated using Fisher's exact or chi-square tests and are presented as counts (percentages). For continuous variables, the Wilcoxon rank-sum test, Student's t-test, or one-way analysis of variance was employed. To explore potential nonlinear relationships between TyG index levels and in-hospital, ICU, and 28-day mortality, a restricted cubic spline analysis was conducted. Four knots were positioned at the 5th, 35th, 65th, and 95th percentiles as recommended by Harrell [21, 22]. To evaluate the association between TyG index and the risk of in-hospital mortality, ICU mortality, and 28-day mortality, multivariate logistic regression analyses were performed. Odds ratios (ORs) and their corresponding 95% confidence intervals (CIs) were calculated to quantify the impact of the TyG index on these outcomes. Model 1 included only the TyG index, without any adjustments. In Model 2, age, Gender and BMI were included as the modified variables. Model 3 incorporated variables based on clinical expertise, prior literature [23], and feature importance selection results from the Boruta and random forest algorithms, as showed in Fig. 2. The variables included in this model were Age; Gender; BMI; SOFA score; Hemoglobin; Sodium; White Blood Cell (WBC); Red Cell Distribution width (RDW); Low-Density Lipoprotein (LDL); Prothrombin Time (PT); Partial Thromboplastin Time (PTT); Alanine Aminotransferase (ALT); Alkaline Phosphatase(ALP); Aspartate Aminotransferase(AST); C-Reactive Protein (CRP); NLR; Atrial Fibrillation; Hypertension; Myocardial Infarction; Congestive Heart Failure (CHF); Chronic Obstructive Pulmonary Disease (COPD); Coronary Artery Disease (CAD); Acute Kidney Injury (AKI); Low HDL. Variance inflation factors (VIFs) were examined to detect multicollinearity among variables. Furthermore, a subgroup analysis was conducted to validate the association between TyG index and in-hospital, ICU, and 28-day mortality within each subgroup. Kaplan–Meier analysis and multivariate Cox regression were also used as sensitivity analyses to explore the association between TyG index and 28-day and 90-day mortality endpoints. In patients with complete SOFA scores, the relationship between TyG index and in-hospital, ICU, and 28-day mortality was performed to affirm the robustness and stability of the obtained results. All statistical analyses were performed using R version 4.1.2 (R Foundation). Statistical significance was defined as a two-sided P-value of < 0.05.

## Results

### Baseline characteristics

The inclusion and exclusion criteria resulted in the enrollment of 1,257 patients with sepsis from the MIMIC-IV database for this study. The in-hospital, 28-day, and ICU mortality were 21.40%, 26.17%, and 15.43% respectively. In Table 1, patients in the TyG T1 group were compared to those in the other groups, revealing that the latter were more likely to be younger and male, with a higher prevalence of obesity, kidney injury, Atrial Fibrillation, Low_HDL. Moreover, patients with a higher TyG index exhibited elevated SOFA score, LDL, Albumin, anion gap, Bicarbonate, blood urea nitrogen, creatinine, potassium, ALT, AST, ALP and NLR levels. Additionally, within the group characterized by a higher TyG index, longer hospital stays and ICU durations were observed. In Table 2, a comparison between the non-hospital survivor and survivor groups revealed that the latter exhibited greater severity of illness, with a higher median SOFA score (10 [IQR, 6–14] vs. 6 [IQR, 4–9]). The results showed that age, Hemoglobin, Platelets, WBC, Mean Cell Volume (MCV), RDW, Total Cholesterol (TC), HDL, LDL, Albumin, Anion gap, Blood Urea Nitrogen (BUN), Bicarbonate, International Normalized Ratio (INR), PT, ALP, AST, Total bilirubin, TyG, NLR were associated with in-hospital mortality. Non-Surviving patients were also more likely to be have a higher prevalence of atrial fibrillation, Low HDL and AKI.

**Table 1:** Characteristics and outcomes of participants categorized by TyG index

TyG index tertile	T1 (8.13–8.91) N = 473	T2 (8.91–9.5)2 N = 415	T3 (9.52–10.96) N = 369	p-value
Demographic				
Age	66.12 (17.73)	62.45 (16.75)	57.90 (16.17)	< 0.001
Gender(male)	259 (54.76%)	226 (54.46%)	230 (62.33%)	0.042
BMI	28.53 (5.95)	29.58 (6.99)	30.89 (6.52)	< 0.001
Laboratory tests				
Hemoglobin (g/dL)	10.93 (2.42)	10.55 (2.46)	10.55 (2.42)	0.029
Platelets (K/uL)	176.00 (126.00–226.00)	185.00 (119.00–237.50)	161.00 (95.00–226.00)	0.005
WBC (K/uL)	12.40 (9.30–16.20)	14.10 (10.50–18.20)	15.80 (11.20–21.30)	< 0.001
MCV (fL)	90.91 (6.63)	90.55 (7.58)	89.70 (7.12)	0.045
RDW (%)	15.19 (2.75)	15.43 (2.63)	15.24 (2.32)	0.345
TC (mg/dL)	143.20 (43.73)	142.27 (42.62)	148.20 (46.81)	0.134
HDL (mg/dL)	46.47 (16.64)	39.83 (14.44)	35.52 (13.01)	< 0.001
LDL (mg/dL)	78.39 (33.25)	77.49 (34.35)	77.50 (34.56)	0.902
Albumin (g/dL)	3.48 (0.56)	3.39 (0.60)	3.20 (0.68)	< 0.001
Anion gap (mEq/L)	16.71 (4.38)	17.88 (4.82)	19.72 (6.48)	< 0.001
Bicarbonate (mEq/L)	24.46 (4.15)	23.79 (4.38)	23.22 (4.94)	< 0.001
BUN (mg/dL)	20.00 (14.00–32.00)	23.00 (17.00–34.00)	27.00 (17.00–45.00)	< 0.001
Calcium (mg/dL)	8.69 (0.84)	8.68 (0.85)	8.57 (0.98)	0.087
Chloride (mEq/L)	105.82 (6.06)	106.79 (6.02)	106.67 (7.40)	0.053
Creatinine (mg/dL)	1.10 (0.80–1.40)	1.20 (0.90–1.80)	1.50 (1.00–2.40)	< 0.001
Sodium (mEq/L)	140.58 (4.77)	141.18 (5.05)	141.08 (6.13)	0.19
Potassium (mEq/L)	4.45 (0.76)	4.63 (0.92)	4.76 (0.89)	< 0.001
Alk. Phosphatase (U/L)	75.11 (62.00–104.04)	76.00 (61.00–103.85)	77.37 (59.00–111.47)	0.062
CRP (mg/dL)	13.70 (4.00–46.70)	40.80 (7.55–111.45)	88.85 (37.72–153.03)	0.002
INR	1.30 (1.10–1.60)	1.30 (1.20–1.65)	1.30 (1.10–1.60)	0.573
PT	4.20 (12.40–17.60)	14.40 (12.80–17.95)	14.20 (12.60–17.70)	0.511
APTT	33.10 (27.80–46.90)	33.30 (28.35–46.70)	32.90 (27.60–51.30)	0.332
ALT (U/L)	31.00 (19.00–78.00)	352.48 (1629.12)	59.00 (29.00–220.00)	0.033
ALP (U/L)	80.00 (66.00–112.90)	83.00 (65.50–118.00)	87.00 (67.00–138.00)	0.004
AST (U/L)	47.00 (27.00–118.00)	68.00 (31.74–184.50)	100.68 (40.00–396.00)	< 0.001
Total Bilirubin (mg/dL)	0.80 (0.54–1.40)	0.80 (0.50–1.60)	0.90 (0.50–1.90)	0.28
NLR	8.43 (4.65–14.87)	9.22 (5.24–15.10)	9.92 (5.86–18.38)	0.019
Comorbidities				
Hypertension	211 (44.61%)	184 (44.34%)	139 (37.67%)	0.084
Myocardial Infarction	95 (20.08%)	104 (25.06%)	75 (20.33%)	0.144
CHF	140 (29.60%)	125 (30.12%)	91 (24.66%)	0.176
COPD	62 (13.11%)	59 (14.22%)	55 (14.91%)	0.748
CAD	108 (22.83%)	111 (26.75%)	76 (20.60%)	0.117
Atrial Fibrillation	179 (37.84%)	122 (29.40%)	87 (23.58%)	< 0.001
Renal Failure	68 (14.38%)	68 (16.39%)	61 (16.53%)	0.617
Chronic Liver Disease	44 (9.30%)	50 (12.05%)	47 (12.74%)	0.236
Stroke	114 (24.10%)	82 (19.76%)	66 (17.89%)	0.071
Malignancy	80 (16.91%)	56 (13.49%)	69 (18.70%)	0.13
Low_HDL	185 (39.11%)	232 (55.90%)	250 (67.75%)	< 0.001
AKI_48hr	290 (61.31%)	299 (72.05%)	285 (77.24%)	< 0.001
SOFA	5.00 (4.00–8.00)	6.00 (4.00–10.00)	8.00 (5.00–12.00)	< 0.001
Organ dysfunction				
Cardiovascular	1.00 (1.00–1.00)	1.00 (1.00–3.00)	1.00 (1.00–4.00)	< 0.001
Neurologic	2.00 (1.00–3.00)	2.00 (1.00–3.00)	2.00 (0.00–3.00)	0.279
Coagulation	0.00 (0.00–1.00)	0.00 (0.00–1.00)	0.00 (0.00–2.00)	< 0.001
Hepatic	0.00 (0.00–1.00)	0.00 (0.00–1.00)	0.00 (0.00–2.00)	0.026
Kidney	0.00 (0.00–1.00)	1.00 (0.00–1.00)	1.00 (0.00–3.00)	< 0.001
Respiratory	2.00 (1.00–3.00)	2.00 (1.00–3.00)	3.00 (2.00–4.00)	< 0.001
Outcomes				
In-hospital mortality	101 (21.35%)	64 (15.42%)	104 (28.18%)	< 0.001
ICU-mortality	68 (14.38%)	43 (10.36%)	83 (22.49%)	< 0.001
28-Day mortality	134 (28.33%)	80 (19.28%)	115 (31.17%)	< 0.001
Icu_Los_Day	4.00 (2.00–8.00)	5.00 (3.00–9.00)	48	0.044
Hospital_Los_Day	10.00 (6.00–16.00)	12.00 (6.00–20.00)	10.00 (5.00–19.00)	0.003

Data: N (%) or Mean (Q1–Q3) or mean ± standard deviation

BMI: Body Mass Index; WBC: White Blood Cell; MCV: Mean cell Volume; RDW: Red Cell Distribution Width; TC: Total Cholesterol; HDL: High-Density Lipoprotein; LDL: Low-Density Lipoprotein; BUN: Blood Urea Nitrogen; Alk. Phosphatase: Alkaline Phosphatase; INR: International Normalized Ratio; PT: Prothrombin Time; APTT: Activated Partial Thromboplastin Time; ALT: Alanine Aminotransferase; ALP: Alkaline Phosphatase; AST: Aspartate Aminotransferase; NLR: Neutrophil-to-Lymphocyte Ratio; CHF: Congestive Heart Failure; COPD: Chronic Obstructive Pulmonary Disease; CAD: Coronary Artery Disease; Low_HDL: Low High-Density Lipoprotein; AKI_48hr: Acute Kidney Injury within 48 h; CRP: C-Reactive Protein; SOFA: Sequential Organ Failure Assessment; ICU_Los_Day: Intensive Care Unit Length of Stay; Hospital_Los_Day: Hospital Length of Stay

**Table 2:** Baseline characteristics of the survivors and non-survivors groups

Categories	Overall N = 1257	Survivor N = 988	Non-survivor N = 269	P-value
Demographic				
Age	62.49 ± 17.27	61.82 ± 17.52	64.94 ± 16.13	0.009
Gender(male)	715 (56.88%)	558 (56.48%)	157 (58.36%)	0.58
BMI	29.57 ± 6.54	29.50 ± 6.32	29.81 ± 7.30	0.49
SOFA	7.44 ± 4.26	6.75 ± 3.76	9.98 ± 4.98	< 0.001
Laboratory tests				
Hemoglobin (g/dL)	10.69 ± 2.44	10.80 ± 2.37	10.29 ± 2.64	0.002
Platelets (K/uL)	182.63 ± 102.33	188.74 ± 102.64	160.20 ± 98.15	< 0.001
WBC (K/uL)	15.57 ± 12.23	14.86 ± 10.07	18.17 ± 17.84	< 0.001
MCV (fL)	90.43 ± 7.11	90.12 ± 6.92	91.60 ± 7.68	0.002
RDW (%)	15.28 ± 2.59	15.09 ± 2.42	15.98 ± 3.04	< 0.001
TC (mg/dL)	144.36 ± 44.34	146.53 ± 44.33	136.38 ± 43.53	< 0.001
HDL (mg/dL)	41.06 ± 15.59	41.83 ± 15.83	38.25 ± 14.34	< 0.001
LDL (mg/dL)	77.83 ± 33.98	78.91 ± 34.43	73.89 ± 32.02	0.032
Albumin (g/dL)	3.37 ± 0.62	3.41 ± 0.59	3.21 ± 0.70	< 0.001
Aniongap (mEq/L)	17.98 ± 5.35	17.25 ± 4.55	20.68 ± 6.98	< 0.001
Bicarbonate (mEq/L)	23.88 ± 4.49	24.33 ± 4.40	22.19 ± 4.44	< 0.001
BUN (mg/dL)	30.19 ± 23.39	28.25 ± 21.91	37.35 ± 27.02	< 0.001
Calcium (mg/dL)	8.65 ± 0.88	8.66 ± 0.81	8.63 ± 1.11	0.66
Chloride (mEq/L)	106.39 ± 6.48	106.34 ± 6.18	106.60 ± 7.51	0.556
Creatinine (mg/dL)	1.68 ± 1.59	1.58 ± 1.60	2.07 ± 1.50	< 0.001
Sodium (mEq/L)	140.93 ± 5.30	140.85 ± 4.94	141.20 ± 6.43	0.331
Potassium (mEq/L)	4.60 ± 0.86	4.55 ± 0.83	4.80 ± 0.93	< 0.001
Alk. Phosphatase (U/L)	100.47 ± 125.44	95.33 ± 102.14	119.33 ± 186.74	0.005
INR	1.62 ± 1.15	1.51 ± 0.93	2.03 ± 1.66	< 0.001
PT	17.78 ± 12.81	16.45 ± 9.88	22.65 ± 19.47	< 0.001
PTT	46.32 ± 32.33	44.54 ± 30.88	52.84 ± 36.51	< 0.001
ALT (U/L)	300.35 ± 1225.85	266.36 ± 1175.83	425.18 ± 1389.52	0.06
ALP (U/L)	114.62 ± 138.79	106.99 ± 112.24	142.65 ± 207.06	< 0.001
AST (U/L)	515.72 ± 1811.76	435.29 ± 1671.11	811.16 ± 2233.23	0.003
Total Bilirubin (mg/dL)	2.27 ± 5.11	1.78 ± 3.78	4.07 ± 8.11	< 0.001
TyG index	9.21 ± 0.66	9.18 ± 0.64	9.33 ± 0.71	< 0.001
CRP (mg/dL)	68.65 ± 73.62	69.90 ± 73.38	63.89 ± 76.24	0.747
NLR	13.22 ± 13.52	12.14 ± 11.52	16.90 ± 18.31	< 0.001
Comorbidities				
Hypertension	534 (42.48%)	425 (43.02%)	109 (40.52%)	0.463
Myocardial Infarction	274 (21.80%)	216 (21.86%)	58 (21.56%)	0.916
CHF	356 (28.32%)	275 (27.83%)	81 (30.11%)	0.462
COPD	176 (14.00%)	135 (13.66%)	41 (15.24%)	0.509
CAD	295 (23.47%)	234 (23.68%)	61 (22.68%)	0.73
Atrial Fibrillation	388 (30.87%)	286 (28.95%)	102 (37.92%)	0.005
Chronic Liver Disease	141 (11.22%)	102 (10.32%)	39 (14.50%)	0.054
Stroke	262 (20.84%)	201 (20.34%)	61 (22.68%)	0.404
Malignancy	205 (16.31%)	158 (15.99%)	47 (17.47%)	0.56
Low HDL	667 (53.06%)	500 (50.61%)	167 (62.08%)	< 0.001
AKI_48 h	874 (69.53%)	650 (65.79%)	224 (83.27%)	< 0.001

Data:N (%) or Mean(Q1-Q3) or Mean ± Standard Deviation

BMI: Body Mass Index; WBC: White Blood Cell; MCV: Mean Corpuscular Volume; RDW: Red Cell Distribution Width; TC: Total Cholesterol; HDL: High-Density Lipoprotein; LDL: Low-Density Lipoprotein; BUN: Blood Urea Nitrogen; Alk. Phosphatase: Alkaline Phosphatase; INR: International Normalized Ratio; PT: Prothrombin Time; APTT: Activated Partial Thromboplastin Time; ALT: Alanine Aminotransferase; ALP: Alkaline Phosphatase; AST: Aspartate Aminotransferase; NLR: Neutrophil-to-Lymphocyte Ratio; CHF: Congestive Heart Failure; COPD: Chronic Obstructive Pulmonary Disease; CAD: Coronary Artery Disease; Low_HDL: Low High-Density Lipoprotein; AKI_48hr: Acute Kidney Injury within 48 h; CRP: C-Reactive Protein; SOFA: Sequential Organ Failure Assessment; ICU_Los_Day: Intensive Care Unit Length of Stay; Hospital_Los_Day: Hospital Length of Stay

### TyG index and in-hospital, ICU, and 28-day mortality

**Fig. 3:**
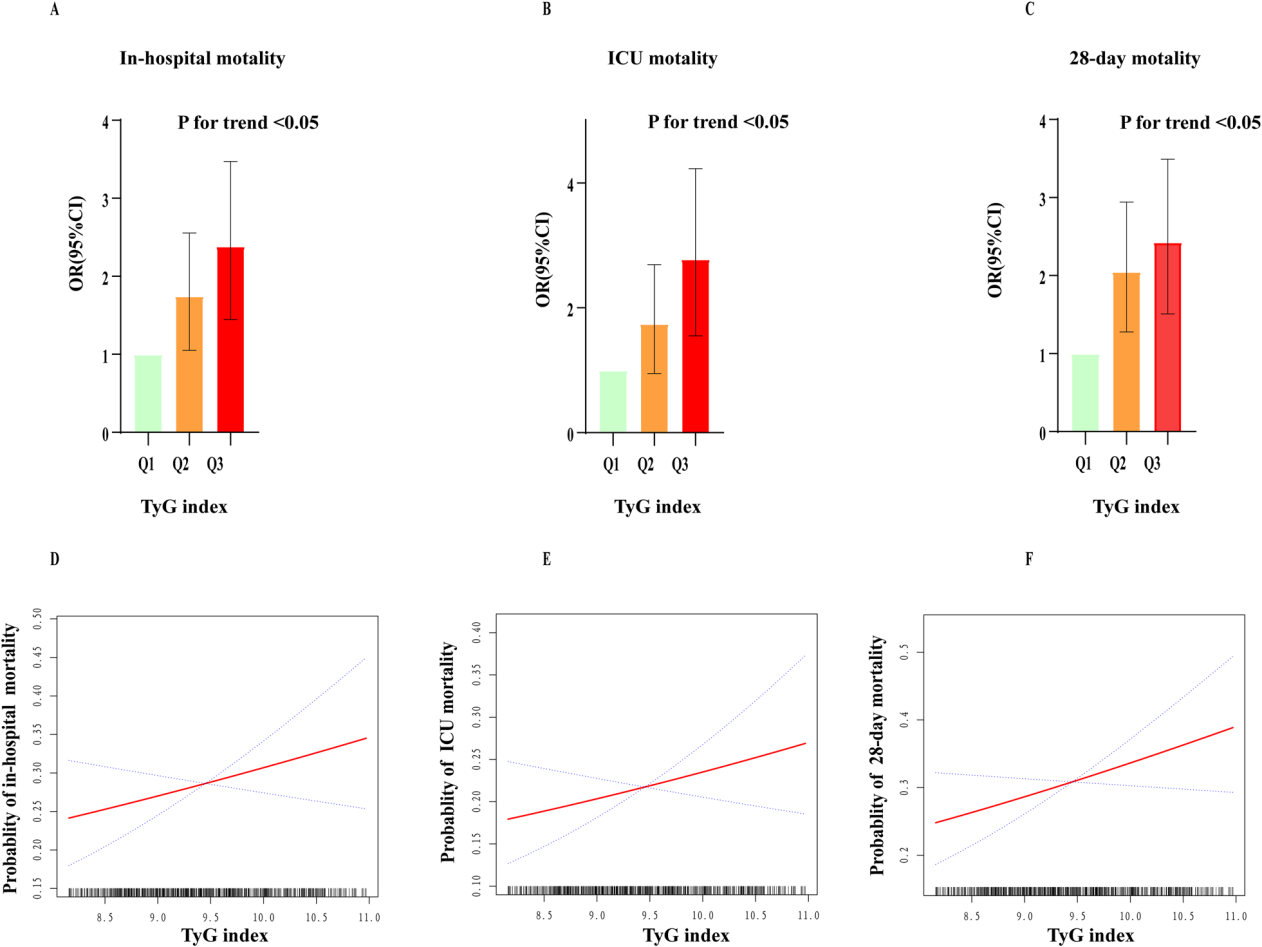
The relationship for the levels of TyG index with in-hospital mortality, ICU mortality, and 28-day mortality. **a**–**c** OR (95% CIs) for in-hospital, in-ICU, and 28-day mortality according to TyG index tertile after adjusting for Adjusted for Age; Gender; BMI; SOFA score; Hemoglobin; Sodium; WBC; RDW; LDL; PT; PTT; ALT; ALP; AST; CRP; NLR; Atrial Fibrillation; Hypertension; Myocardial Infarction; CHF; COPD; CAD; AKI; Low HDL. Error bars indicate 95% CIs. The first tertile is the reference. **d** Restricted cubic spline for hospital mortality. **e** Restricted cubic spline for ICU mortality. **f** Restricted cubic spline for 28-day mortality. OR: odds ratio; CI: confidence interval; ICU: intensive care unit; TyG: triglyceride-glucose

Multivariate logistics regression analysis showed that the TyG index was independently associated with an elevated risk of in-hospital mortality (OR 1.440 [95% CI 1.106–1.875]; P = 0.00673), 28-day mortality (OR 1.391; [95% CI 1.52–1.678]; P = 0.01414) and ICU mortality (OR 1.597; [95% CI 1.188–2.147]; P = 0.00266). These results were further confirmed in the fully adjusted Model 3, specifically, the OR for in-hospital mortality in the highest TyG index tertile was 2.242, 95% CI: 1.448–3.472, and for ICU mortality, it was 2.564, 95% CI: 1.553–4.233, both compared with the lowest tertile. Additionally, the risk of in-hospital mortality, ICU mortality, and 28-day mortality demonstrated a consistent upward trend with increasing TyG index tertiles, with all trend p-values below 0.05 (Table 3). Moreover, the restricted cubic spline regression model was applied to reveal that the risks of in-hospital mortality, ICU mortality, and 28-day mortality increased linearly with increasing TyG index (Fig. 3). For stability of results, the Kaplan–Meier analysis plot showed a significant difference among various TyG index groups of 28-day and 90-day mortality (Additional file 1: Fig. S1). Among patients with complete components of the SOFA score, the relationship between the TyG index group and in-hospital, ICU, and 28-day mortality was consistent with core results (Additional file 2: Table S2) and the association between TyG index groups and mortality in patients with sepsis for Cox Regression is stable (Additional file 2: Table S3).

**Table 3:** The association between TyG index groups and in-hospital, ICU and 28-day mortality

Exposure	Model 1		Model 2		Model 3	
	OR (95% CI)	P-value	OR (95% CI)	P-value	OR (95% CI)	P-value
In-hospital mortality						
TyG as continuous	1.417 (1.160, 1.730)	0.00064	1.520 (1.233, 1.875)	0.00009	1.440 (1.106, 1.875)	0.00673
Q1	Ref		Ref		Ref	
Q2	1.489 (1.054, 2.103)	0.02389	1.427 (1.007, 2.022)	0.04562	1.638 (1.050, 2.556)	0.02976
Q3	2.152 (1.517, 3.054)	0.00002	2.271 (1.592, 3.240)	< 0.00001	2.242 (1.448, 3.472)	0.0003
P for trend	0.00002		< 0.00001		0.00029	
ICU mortality						
TyG as continuous	1.598 (1.276, 1.999)	0.00004	1.627 (1.287, 2.056)	0.00005	1.597 (1.188, 2.147)	0.00195
Q1	Ref		Ref		Ref	
Q2	1.453 (0.967, 2.182)	0.0722	1.435 (0.952, 2.163)	0.08441	1.597 (0.946, 2.695)	0.07954
Q3	2.511 (1.684, 3.743)	< 0.00001	2.525 (1.686, 3.781)	< 0.00001	2.564 (1.553, 4.233)	0.00023
P for trend	< 0.00001		< 0.00001		0.00021	
28-day mortality						
TyG as continuous	1.222 (1.013, 1.476)	0.03652	1.416 (1.159, 1.729)	0.00066	1.371(1.066,1.764)	0.01414
Q1	Ref		Ref		Ref	
Q2	1.655 (1.207, 2.269)	0.00174	1.533 (1.112, 2.114)	0.00906	1.938 (1.277, 2.942)	0.00187
Q3	1.896 (1.365, 2.634)	0.00014	2.164 (1.543, 3.033)	< 0.00001	2.295 (1.507, 3.493)	0.00011
P for trend	0.00014		< 0.00001		0.0001	

Model 1: Unadjusted

Model 2: Adjusted For Age; Gender; BMI

Model 3: Adjusted For Age; Gender; BMI; SOFA score; Hemoglobin; Sodium; WBC; RDW; LDL; PT; PTT; ALT; ALP; AST; CRP; NLR; Atrial Fibrillation; Hypertension; Myocardial Infarction; CHF; COPD; CAD; AKI; Low_HDL

### Subgroup analysis

**Fig. 4:**
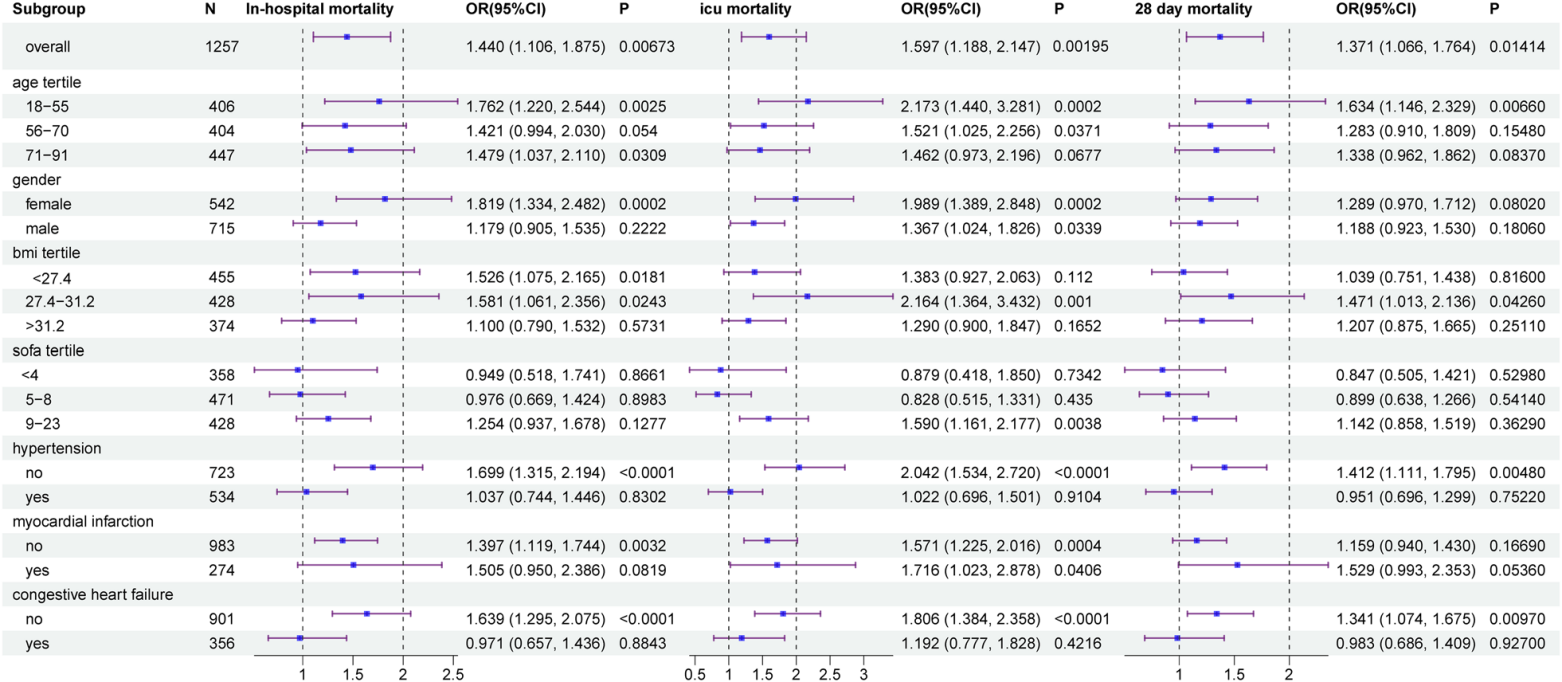
Subgroup analyses for the association of TyG index with in-hospital mortality, ICU mortality, and 28-day mortality. OR: odds ratio, CI: confidence interval

Furthermore, to confirm the relationship between TyG index and in-hospital mortality, ICU mortality, and 28-day mortality, stratified analyses were conducted based on age, Gender, BMI, SOFA score, hypertension, myocardial infarction, and congestive heart failure. In Fig. 4, There is a significant relationship between TyG and ICU mortality for both males (OR = 1.367, 95% CI 1.024–1.826) and females (OR = 1.989, 95% CI 1.389–2.848) in full adjusted model, as well as for individuals with myocardial infarction (OR = 1.571, 95% CI 1.225–2.016) and those without myocardial infarction (OR = 1.716, 95% CI 1.023–2.878). The relationship between the TyG index and mortality remains consistent in direction. Regardless of the outcome variable being in-hospital mortality, ICU mortality, or even 28-day mortality, the results of the stratified analysis consistently demonstrated a similar association of TyG index values across most sub-populations.

## Discussion

To our knowledge, this study represents the first investigation into the relationship between the TyG index and all-cause mortality among sepsis patients. In this study, we found a significant association between an elevated TyG index and increased in-hospital mortality among patients with sepsis. This conclusion aligns with ICU and 28-day mortality in patients with sepsis, which shown a consistent linear relationship. This association remained robust, even after adjusting for multiple clinical and laboratory variables. Our results extended the application of the TyG index to the realm of cardiovascular disorders, indicating its potential value as a decision-making tool for clinicians managing patients with sepsis.

In recent years, TyG index has been proposed as a potential marker for metabolic disorders, atherosclerotic conditions, cardiovascular disease and COVID-19 [24–27]. Numerous clinical studies have examined the association between an elevated TyG index and higher morbidity and mortality associated with critically ill patients and infectious diseases across various populations. Yang et al. found that the TyG index was an independent risk factor for in-hospital and ICU mortality in patients after cardiac arrest [7]. Lee et al. observed that the TyG index might be helpful in predicting short-term functional outcomes in critically ill stroke patients undergoing reperfusion therapy [28]. Additionally, for individuals with coronary artery disease, the TyG index could contribute to predicting adverse cardiovascular events [29, 30]. For general population, a high TyG index has been found to be associated with an increased incidence of respiratory symptoms, an elevated risk of chronic lung disease, and a reduction in lung function [31]. These studies collectively suggest the potential of the association between TyG index and clinical outcomes in critically ill and infection-related patients. Other studies have reported that for each unit increase in the TyG index, the risk of in-hospital mortality in critically ill patients increases by nearly 30% or more [8, 9]. We observed that for each unit increase in the TyG index, the risk of in-hospital mortality for sepsis patients increased by 44.0%, our study's conclusions align with previous researches, indicating an association between elevated TyG index and increased mortality.

Our results suggest an association between a high TyG index and the severity and outcomes of sepsis. Sepsis can lead to insulin resistance and the disruption of lipid metabolism accompanied by uncontrolled hyperglycemia and glycemic variability during the acute phase of sepsis. The prognosis of sepsis is closely tied to the severity of inflammatory responses, which are significantly correlated with insulin resistance. Our results demonstrate that the TyG index is positively correlated with disease severity scores. The TyG index reflects the severity of disease in patients with sepsis and provides insights that can contribute to the clinical management of sepsis. Clinicians should be aware of patient blood glucose management and monitor changes in insulin resistance indicators.

The exact biological mechanisms underlying the relationship between the TyG index and sepsis prognosis remain unclear. The TyG index is associated with insulin resistance (IR), insulin resistance has been widely demonstrated to be closely related to endothelial dysfunction, oxidative stress, immune dysregulation, coagulation imbalance, and inflammatory response [32–34], all of which are also closely associated with the occurrence and progression of sepsis. From the baseline data, we observed significant differences in SOFA scores among patients in different TyG index groups, indicating a close association between the TyG index and disease severity. Changes in insulin resistance during the acute phase of sepsis could reflect the inflammatory state or severity of sepsis. A potential explanation for the role of the TyG index as an indicator of cardiovascular disease could be that the TyG index serves as a reflection of IR in patients. IR, in turn, can contribute to the development of cardiovascular diseases by enhancing vascular stiffness and diminishing the bioavailability of nitric oxide (NO) [35, 36]. An elevated TyG index is associated with cardiovascular diseases, and the presence of cardiovascular diseases in sepsis is a risk factor contributing to adverse patient outcomes. Although cardiovascular diseases were present in the various TyG index level groups in this study, no differences were observed.

In sensitivity analysis, our study found that the linear relationship between the TyG index and in-hospital mortality in sepsis patients remained consistent in the younger age group, female patients, those with lower BMI, non-hypertensive individuals, and those without congestive heart failure. This result might be attributed to the fact that advanced age, male, higher BMI, and hypertension are traditionally recognized as unfavorable risk factors for sepsis prognosis. Furthermore, factors such as Gender, obesity, and cardiovascular disease can contribute to insulin resistance, potentially leading to an underestimation of the association between the TyG index and sepsis outcomes. Meanwhile, in subgroup analyses and regarding the 28-day mortality and ICU mortality, we observed that after stratifying by SOFA score and gender, the results were not statistically significant. After stratified analysis, the sample size decreases, leading to a reduction in effect size, which can be one of the reasons for non-significant results. However, the consistent direction of all results indicates the stability and reliability of the core outcomes. In patients with complete SOFA scores, the relationship between the TyG index group and in-hospital ICU, and 28-day mortality remained stable. In Cox regression analysis, the relationship between 28-day and 90-day mortality rates in the TyG index group was consistent with core results. Furthermore, we performed a feature analysis utilizing SHAP and Brouta plots, and the importance of the TyG index as a feature was evaluated within the outcome prediction model. Consequently, a machine learning prognostic model for sepsis could be established in the future by focusing on the TyG index.

However, it is important to acknowledge the limitations of this study. First, our analysis was retrospective and derived from observational data, precluding the definitive establishment of causality. Nonetheless, we employ a range of rigorous statistical methods to yield robust and credible outcomes. Second, the TyG index was not dynamically monitored, and sepsis itself could influence lipid metabolism and blood glucose fluctuations. The TyG index obtained from the first-time glucose and triglyceride measurements may not comprehensively represent insulin resistance in the body. Third, some confounding factors, including metabolic syndrome parameters, Acute Physiology and Chronic Health Evaluation II (APACHE II), nutritional state parameters, and inflammatory markers, were not thoroughly considered. This may have an impact on the results. Fourth, clinicians were following different guidelines with different definitions of sepsis from 2008 to 2019, which could potentially impact the study results. Fifth, when patients receive enteral or parenteral nutrition, it may impact lipid and glucose levels, potentially leading to an increase in the TyG index. However, with the large sample size included in our study, this effect is likely mitigated. Further research is needed to investigate the key mechanisms of insulin resistance in patients with sepsis.

## Conclusions

The elevated TyG index is strongly associated with increased in-hospital all-cause mortality in patients with sepsis. Our results suggest that the TyG index assists in the early detection of insulin resistance in patients with sepsis, thus enhancing risk assessment and directing subsequent interventions. However, additional prospective studies are required to validate these findings.

### Supplementary Information

**Additional file 1:** Additional methods. **Figure1S:** The Kaplan–Meier analysis plot showed a significant difference among various TyG index groups of 28-day mortality (A.B) and 90-day mortality (C.D).

**Additional file 2: Table S1.** Each component of the SOFA score categorized by TyG index. **Table S2.** The association between TyG index groups and in-hospital, ICU and 28-day mortality in patient with all component of the SOFA score (n = 733). **Table S3.** The association between TyG index groups 28-day and 90-day mortality for Cox Regression. **Table S4.** The association between TyG index on 28-day and 90-day mortality for Cox Regression in patient with all component of the SOFA score (n = 733). **Table S5.** Missing rate for demographics and clinical variables extracted from the database during the observation period. **Table S6.** Subgroup analyses for the association of TyG index with in-hospital death, ICU death, and 28-day death.

## Data Availability

Raw data supporting the obtained results are available at the corresponding author.
