# Supplementary material for: Association between triglyceride-glucose index and in-hospital mortality in critically ill patients with sepsis: analysis of the MIMIC-IV database
Source: Cardiovasc Diabetol. 2023 Nov 8;22:307. doi: 10.1186/s12933-023-02041-w (PMC10634031; doi:10.1186/s12933-023-02041-w)

**Additional methods:**

**Patients with Sepsis3.0 in MIMIC database**

Sepsis was defined based on previously described methods, including (1) Clinical suspicion of infection as determined by the earlier timestamp of antibiotics administration, and cultures within a certain timeframe. If antibiotics were given first, then the cultures must have been obtained within 24 hours. If cultures were obtained first, then antibiotics must have been subsequently ordered within 72 hours. (2) The occurrence of end-organ damage as identified by a two-point deterioration in SOFA score. (3) The onset time of sepsis is the earlier of $t_{\mathrm{suspicion}}$ and $t_{\mathrm{SOFA}}$ as long as $t_{\mathrm{SOFA}}$ occurs no more than 48 hours before or 24 hours after $t_{\mathrm{suspicion}}$; otherwise, the patient is not marked as a sepsis patient. Specifically, if $t_{\mathrm{suspicion}}$ -48 ≤ $t_{\mathrm{SOFA}}$ ≤ $t_{\mathrm{suspicion}}$ +24, then $t_{\mathrm{sepsis}}$ = min ($t_{\mathrm{suspicion}}$, $t_{\mathrm{SOFA}}$) [1; 2]. The Structured Query Language (SQL) code used for data extraction can be found at [mimic-iv/concepts/sepsis at master · MIT-LCP/mimic-iv · GitHub](https://github.com/MIT-LCP/mimic-iv/tree/master/concepts/sepsis).

1. **Approach to missing data**

Missing data were summarized in Tables S5. No consensus exists regarding the standard percentage of missing values for excluding a variable from analysis. For our study, we have elected to set our threshold at 60%, bearing in mind that Zhang et al. [3] have omitted variables with over 70% missing values in their analysis. Before each model fitting process, we assumed missing data were “missing at random” (MAR) [4; 5]. The *“*missForest*”* package in R studio was employed to impute the data [6; 7].

**Reference:**

[1] M. Yang, C. Liu, X. Wang, Y. Li, H. Gao, X. Liu, and J. Li, An Explainable Artificial Intelligence Predictor for Early Detection of Sepsis. Crit Care Med 48 (2020) e1091-e1096.

[2] M.A. Reyna, C.S. Josef, R. Jeter, S.P. Shashikumar, M.B. Westover, S. Nemati, G.D. Clifford, and A. Sharma, Early Prediction of Sepsis From Clinical Data: The PhysioNet/Computing in Cardiology Challenge 2019. Crit Care Med 48 (2020) 210-217.

[3] Z. Zhang, K.M. Ho, and Y. Hong, Machine learning for the prediction of volume responsiveness in patients with oliguric acute kidney injury in critical care. Crit Care 23 (2019) 112.

[4] M.E. Montez-Rath, W.C. Winkelmayer, and M. Desai, Addressing missing data in clinical studies of kidney diseases. Clin J Am Soc Nephrol 9 (2014) 1328-35.

[5] J.D. Dziura, L.A. Post, Q. Zhao, Z. Fu, and P. Peduzzi, Strategies for dealing with missing data in clinical trials: from design to analysis. Yale J Biol Med 86 (2013) 343-58.

[6] Z. Zhang, Multiple imputation with multivariate imputation by chained equation (MICE) package. Ann Transl Med 4 (2016) 30.

[7] D.J. Stekhoven, and P. Buhlmann, MissForest--non-parametric missing value imputation for mixed-type data. Bioinformatics 28 (2012) 112-8.

**Figure1S: The Kaplan–Meier analysis plot showed a significant difference among various TyG index groups of 28-day mortality (A.B) and 90-day mortality (C.D)**


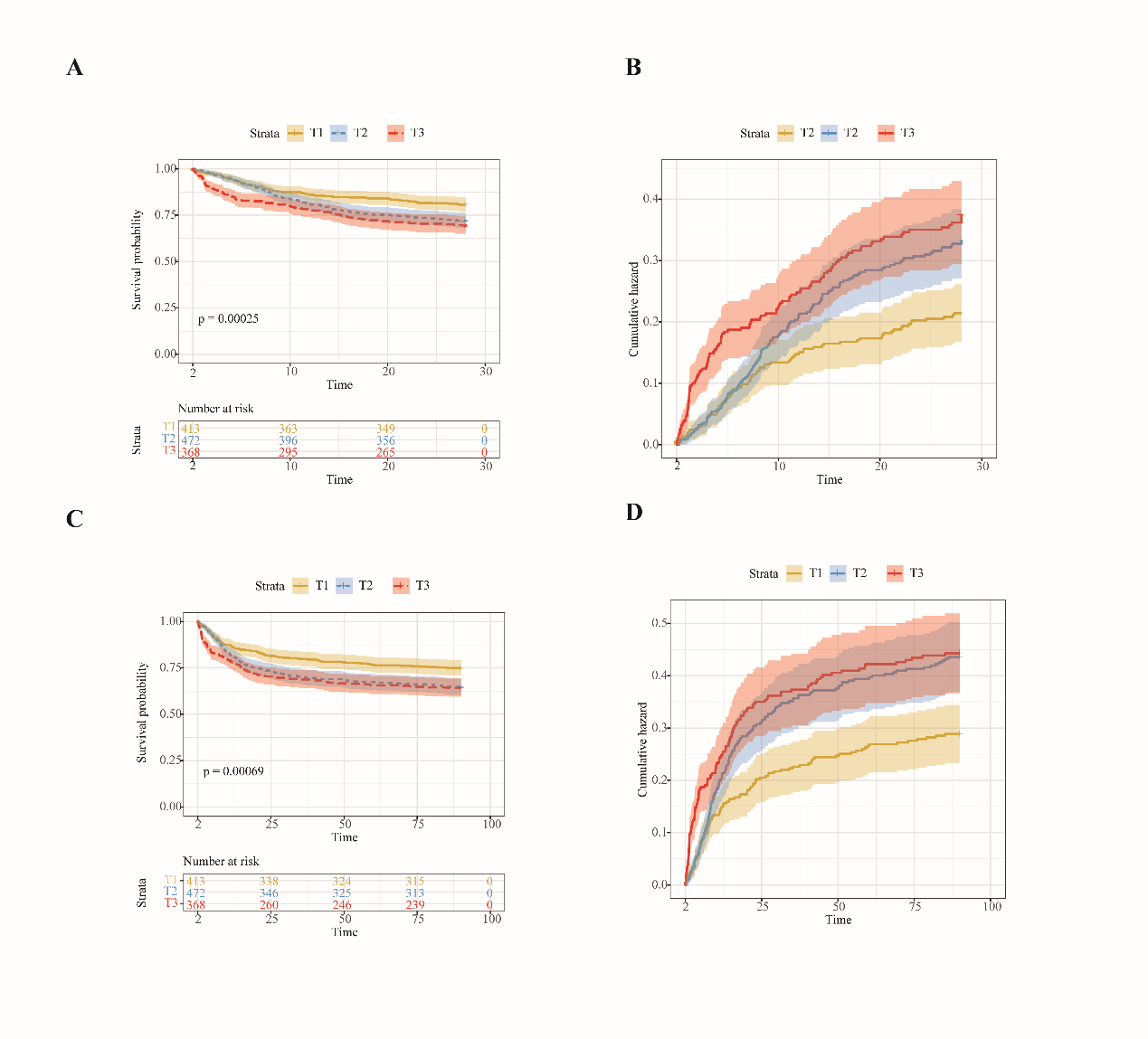

Supplement: Supplementary file 1 — Additional file 1: Additional methods. Figure1S: The Kaplan–Meier analysis plot showed a significant difference among various TyG index groups of 28-day mortality (A.B) and 90-day mortality (C.D). [file 12933_2023_2041_MOESM1_ESM.docx]
